# Ionophore-Based Voltammetric Ion Transfer Microscopy: Imaging Enzyme-Generated Ammonium in Flow

**DOI:** 10.1021/acsmeasuresciau.6c00226

**Published:** 2026-09-11

**Authors:** Justine A. Rothen, Eric Bakker

**Affiliations:** Department of Inorganic and Analytical Chemistry, 27212 University of Geneva, Geneva 1211, Switzerland

**Keywords:** voltammetric ion transfer microscopy, electrochemical imaging, quantitative mapping, ionophore, ion-selective membrane

## Abstract

Spatially resolved measurements of ion concentration gradients provide valuable insight into chemical, biological, and environmental processes. Recently, Voltammetric Ion Transfer Microscopy (VITM) was introduced as a novel opto-electrochemical imaging technique capable of rapidly mapping ionic species across a large (>mm^2^) surface at high spatial resolution. Its working principle is based on electrochemically driven ion transfer across a thin membrane coupled to the modulation of a fluorescent signal. Local ion concentration changes at the membrane are visualized by a shift in the potential (and therefore acquisition time) at which the fluorescence change occurs. Although this technique allows for the acquisition of millions of chemical data points within seconds, its analytical applicability has so far been limited to lipophilic species in the Hofmeister selectivity sequence. Here, we report the first ionophore-based implementation of VITM, demonstrating a general strategy to overcome this limitation by the incorporation of an ionophore into the sensing membrane. This introduces selective chemical recognition while preserving the fundamental sensing mechanism of VITM. Ammonium was selected as a model ion by using a recently introduced tripodal ionophore containing triazole groups as hydrogen bond-forming recognition elements. Simultaneous electrochemical and optical selectivity measurements demonstrate that the optical response faithfully reproduces the electrochemical selectivity, giving a two-orders-of-magnitude preference for ammonium over potassium ions. As a proof of concept, the methodology was applied to visualize ammonium generated *in situ* by hydrolysis of urea catalyzed by jack bean urease at a microfluidic flow junction. The resulting concentration maps reveal the formation of an ammonium concentration gradient that shows excellent agreement with simulations realized for the same experimental conditions. The proposed strategy should be adaptable to other target ions by the incorporation of appropriate selective ionophores, thereby extending the analytical applicability of VITM.

## Graphical abstract

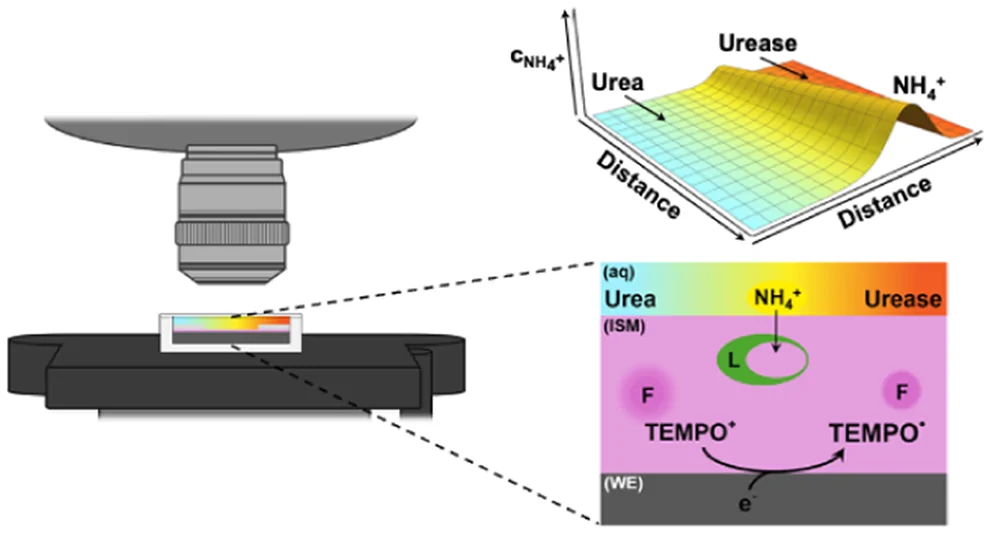

## Introduction

Spatial concentration gradients of ionic species play a key role in numerous chemical, biological and environmental processes. Mapping these heterogeneous distributions provides valuable insight of interfacial phenomena, mass transport, and catalytic (including enzymatic) activity that often determine macroscopic behavior. Consequently, considerable effort has been devoted to the development of analytical techniques capable of mapping distributions of ionic species.^1,2^ For the detection of optically silent species, scanning probe techniques are probably the most established methods for obtaining spatially resolved chemical information. In particular, Scanning Electrochemical Microscopy (SECM), pioneered by Bard and coworkers, has become a reference technique for probing local ionic activity with high sensitivity and spatial resolution.^3^ Nevertheless, SECM and related scanning probe techniques rely on sequential point-by-point measurements. Besides needing dedicated instruments, this limits acquisition speed, as the total imaging time increases proportionally with both image size and spatial resolution.^4^ Scanning probes may also be regarded as invasive when probing solution gradients because the presence of the moving probe will perturb the system under study.^5^

To address these limitations, our group recently introduced Voltammetric Ion Transfer Microscopy, a new electrochemical imaging approach based on dynamic electrochemistry that does not incorporate any moving parts. Its principle relies on an electrochemically driven ion transfer mechanism across a thin ion-selective membrane that is coupled to a modulation of a fluorescent signal. The acquisition time (and therefore potential) during the voltage scan at which the largest fluorescence change occurs for each pixel serves as signal. This information is converted to an ion activity map by applying the Nernst equation. Instead of scanning a probe over the surface, the entire sensing area is interrogated simultaneously. In a voltammetric microscopy experiment, each pixel of the recorded fluorescence image sequence behaves as an individual sensing element, allowing millions of localized optical measurements correlated to the electrochemical perturbation to be acquired in parallel within a few seconds. This makes VITM attractive for investigating dynamic chemical systems involving ionic species.^6^

Despite this advantage, the first generation of VITM exhibited limited chemical selectivity that was governed by the intrinsic Hofmeister selectivity sequence. In this case, ion transfer is mainly dictated by the relative lipophilicity of the ions and their hydration energy, resulting in a predictable but nonspecific selectivity pattern.^7^ While this behavior was sufficient to establish the fundamental working principle of the technique, the lack of ion specific recognition considerably restricts the analytical applicability of VITM in more complex chemical or biological environments where multiple competing ionic species can be present.

A well-established strategy to introduce chemical selectivity in ion-responsive membranes relies on the use of ionophores, which are lipophilic ion-selective complexing agents that can be dissolved in polymeric membranes.^8,9^ While being extensively employed in potentiometric ion-selective electrodes and in ion transfer voltammetry, their integration into VITM has yet to be established.

In this work we demonstrate that the incorporation of an ionophore into the VITM sensing membrane allows for selective ion imaging while preserving the fundamental opto-electrochemical mechanism of the technique. As a proof of concept, ammonium was selected as model ion due to its relevance in chemical and biological systems.^10–12^ The chosen ionophore is a recently reported 6-fold substituted tripodal benzene derivative (see structure in Figure 1) that forms 1:1 complexes with ammonium ions. The introduction of three *n*-hexyl chains endows the ionophore with adequate lipophilicity, which minimizes ionophore leaching from the membrane.^13^

**Figure 1:**
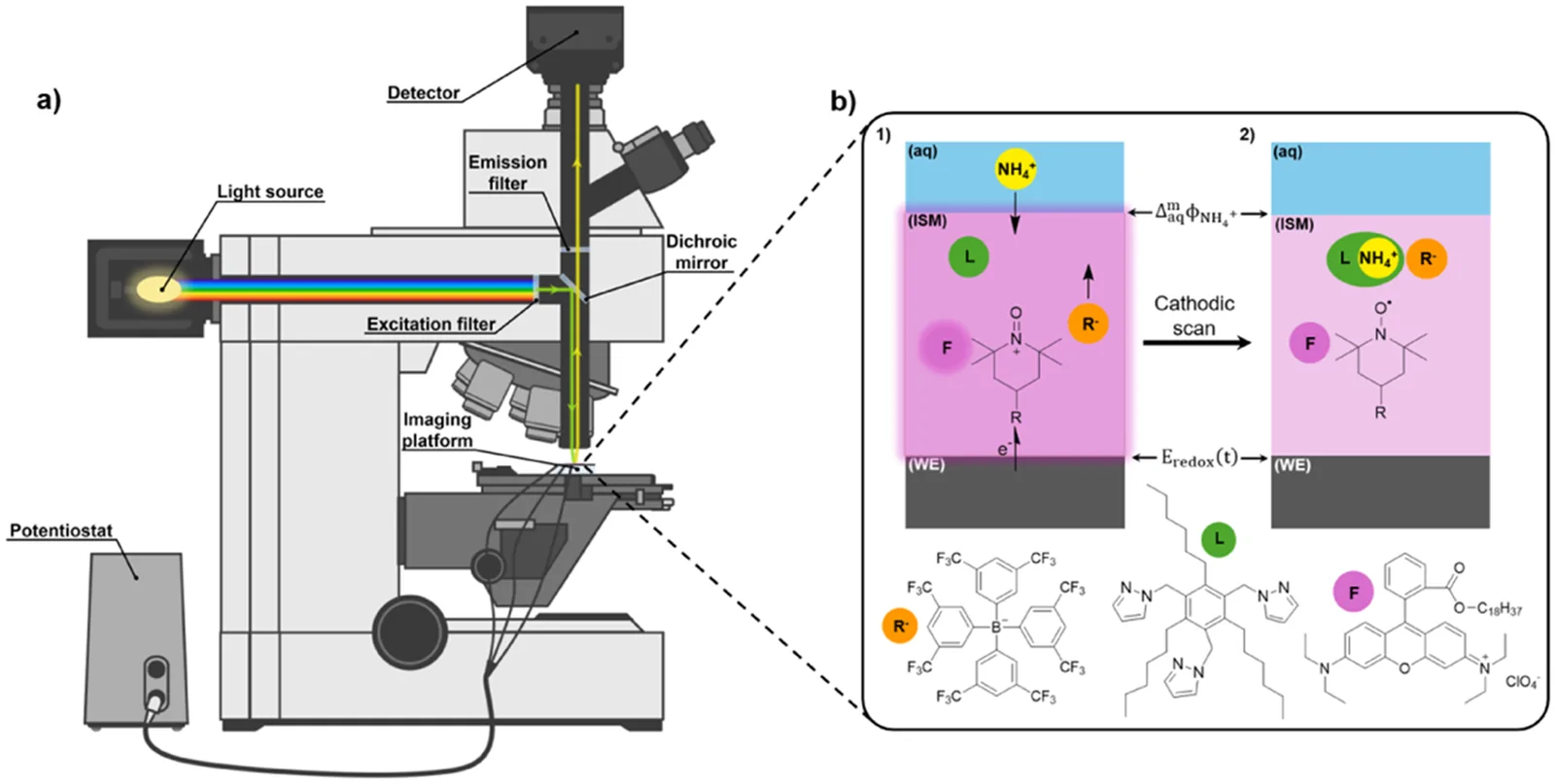
(a) Schematic representation of the opto-electrochemical experimental setup. The imaging platform is placed on a microscope stage and connected to a potentiostat. A high-resolution image stack is acquired while applying a linear sweep voltammetry protocol to record the change of fluorescence intensity of the membrane deposited on the working electrode (WE). (b) Underlying opto-electrochemical sensing mechanism. The thin polymeric membrane (ISM) incorporates a redox probe (lip-TEMPO, R represents a heptadecyl ester), a cation-exchanger (R^–^), a lipophilic fluorophore (F) and the ammonium selective ionophore (L). A linear potential sweep converts the lip-TEMPO from its cationic form into its radical form. To maintain membrane electroneutrality imposed by R^–^, this results in the transfer of an ammonium ion from the aqueous solution in the organic phase. Simultaneously, the fluorescence of F is quenched by the presence of the radical form of the redox probe lip-TEMPO^•^. The image sequence serves to capture the potential of maximum fluorescence change, which is then used to construct the associated peak potential and concentration map.

The approach was investigated with the combined electrochemical and optical readout of VITM, allowing for the first time to simultaneously determine electrochemical and optical selectivity coefficients. The capabilities of the method are demonstrated by visualizing ammonium generated by the enzymatic activity of jack bean urease in a microfluidic device containing a flowing junction, allowing direct observation of the spatial development of the concentration gradient during the reaction.

## Experimental Section

### Chemicals and Instrumentation

Ammonium nitrate (NH_4_NO_3_, ≥99%), magnesium chloride (MgCl_2_, ≥98%), potassium nitrate (KNO_3_, ≥99%), calcium nitrate tetrahydrate (Ca­(NO_3_)_2_ · 4H_2_O, ≥99%), sodium tetrakis­[3,5-bis­(trifluoromethyl)­phenyl]-borate (NaTFPB, R^–^), high molecular weight poly­(vinyl chloride) (PVC), bis­(2-ethylhexyl)­sebacate (DOS, ≥97%), tetrahydrofuran (THF), carbon nanotubes (single-walled, octadecylamine functionalized, 80–90% carbon basis, D × L 2–10 nm × 0.5–2 μm, bundle dimensions), sodium hydroxide (NaOH, 98.5%), 3-(N-morpholino)­propanesulfonic acid (MOPS, ≥99.5%) and bovine serum albumin (BSA, ≥96%) were purchased from Sigma-Aldrich, Merck, Germany. Rhodamine B octadecyl ester perchlorate (lip-RhB, F) was sourced from Chemodex, Switzerland. Sodium nitrate (NaNO_3_, ≥99%), lithium nitrate (LiNO_3_, 99%) and urea (99%) were ordered from Thermo Fisher Scientific, Switzerland. 1-(Dodecyloxy)-2-nitrobenzene (NPOE) was obtained from BLD Pharm, Germany. Urease from jack bean was acquired from Serva, Chemie Brunschwig AG. The tripodal ammonium selective ionophore (L) was provided by Eaglenos, Switzerland. 1-Oxyl-2,2,6,6-tetramethylpiperidin-4-yl stearate (lip-TEMPO) was prepared according to the synthesis described by Mattos et al.^14^ All chemicals were used as received without any prior additional treatment. Aqueous solutions were all prepared in Milli-Q water (>18.2 MΩ cm specific resistance). Ag/AgCl-coated wires, glassy carbon electrodes and a double-junction Ag/AgCl|3 M KCl|1 M LiOAc reference electrode from Metrohm, Switzerland, were used. Square glassy carbon plates (25 mm × 25 mm × 1 mm) were acquired from Hochtemperatur Werkstoffe GmbH, Germany. Voltammetric measurements were performed using a PGSTAT101 (Metrohm Autolab, The Netherlands), controlled by NOVA 2.1 software. Potentiometric measurements were recorded with an EMF16 Interface (Lawson Laboratories, Inc., Malvern, PA). Fluorescence images were captured using an Axio Imager Z1m widefield fluorescence microscope (Zeiss, Germany) equipped with a high-resolution, fast and sensitive sCMOS monochrome camera (Hamamatsu, ORCA-Flash 4.0LT).

### Preparation of the Imaging Platform

Prior to membrane deposition, the square glassy carbon plate serving as working electrode (WE) was polished sequentially using diamond powder suspensions of decreasing particle sizes (Ø 6, 3, 1, and 0.25 μm).

The ion-selective membrane cocktail was composed of a redox probe (lip-TEMPO), a cation-exchanger (NaTFPB) and an ammonium-selective ionophore at equimolar concentrations (50 mmol kg^–1^), together with a lipophilic fluorophore (lip-RhB, 21 mmol kg^–1^). These components were incorporated into a polymeric matrix based on PVC and the plasticizer DOS at a ratio of 1:3 (w/w). A total mass of 50 mg was dissolved in 1 mL THF.

Subsequentially, the thin membrane was coated on the WE by drop-casting 25 μL of the membrane cocktail onto the electrode surface in a spinning apparatus (Lab Spin, Süss Microtech) at 1000 rpm for 60 s to evaporate the solvent and thus form a uniform thin film. Based on previous work, the membrane thickness was estimated to be approximately 230 nm.^6^

To delimit the electrode area, a transparent tape (3M Transpore Professional care, 100 μm thickness for measurements in the regular electrochemical imaging cell (Figure S3) or Scotch 3M, crystal clear, 50 μm thickness in the electrochemical imaging flow cell (Figure S8) with a perforated circular opening was placed on top of the membrane.

The modified WE was then assembled in the electrochemical imaging cell with an Ag/AgCl-coated wire and a platinum wire serving respectively as reference (RE) and counter electrodes (CE).

Both the regular electrochemical imaging cell and flow cell were designed and fabricated in-house and are directly compatible to be used with commercially available upright fluorescence microscopes. The flow cell is described in greater detail in previous work.^6^

For experiments performed with urease, the ISM was protected with a 3% (w/w) BSA solution prior to closing the flow cell to prevent enzyme adsorption on the surface. This precaution was necessary because preliminary experiments showed that the enzyme lost its activity when directly placed on the membrane. This may be attributed to the presence of the TEMPO derivative in the membrane, which has been reported to readily oxidize amine groups.^15,16^

### Opto-Electrochemical Measurements

The imaging platform was placed on the stage of an upright fluorescence microscope for measurements. A linear cathodic potential scan was applied over a 300 mV window at a scan rate of 15 mV s^–1^ for a total acquisition time of 20 s. The scan rate was adopted from our previous work to provide an appropriate compromise between potential resolution of the optical readout, fluorescence signal intensity and electrochemical current.^6^ Additionally, under these conditions, the sensing membrane follows thin-layer behavior.^17^ Simultaneously, an image sequence of the ion-selective membrane was captured through a 5x objective. The fluorescence images were acquired at a frequency of 8.35 frames s^–1^ with an exposure time of 120 ms per frame. A fluorescence filter cube appropriate for rhodamine dyes was used. An in-house built hardware circuit was used to trigger synchronized electrochemical and optical data acquisition.

### Image Analysis Procedure

The automated image analysis procedure applied to treat optical data was identical to that described in previous work^6^ except that it was adapted to identify the maximum fluorescence intensity change associated with the cathodic, rather than anodic peak.

## Results and Discussion

### Opto-Electrochemical Sensing Mechanism

In the present work, the previously reported opto-electrochemical imaging platform was adapted for the selective detection of ammonium by incorporating a recently introduced tripodal ammonium ionophore into the sensing membrane.^6,13^ This modification does not affect the fundamental working principle of VITM, which relies on thin-layer conditions that ensure rapid equilibration of the ion-transfer process during the potential scan. The sensing membrane also contained a lipophilic TEMPO derivative (lip-TEMPO) as redox probe and a lipophilic rhodamine B derivative as the optical reporter (see structures in Figure 1), which follows the previous experimental protocol.^6^

Previous electrochemical characterization of this ionophore by cyclic voltammetry has shown that the cathodic wave exhibited a response closer to Nernstian behavior than the corresponding anodic wave.^13^ It was therefore expected to provide improved optical response and was thus chosen against the anodic wave. This modification reverses the direction of the optical signal compared to the protocol using the anodic wave, so the image analysis routine was adapted accordingly.

The underlying mechanism is schematically shown in Figure 1b. When a linear cathodic potential scan is applied, the oxoammonium form of lip-TEMPO is reduced to its stable nitroxide radical form in a one-electron process.^14,18^ Because the radical species is electrically neutral, it no longer compensates the charge of the lipophilic cation exchanger present in the membrane. To preserve the electroneutrality of the organic phase, a cation from the aqueous phase is transferred into the membrane. Under ideal thin-layer equilibrium conditions, the total charge transferred is therefore determined by the amount of cation-exchange sites in the membrane and is essentially independent of the ion concentration in the aqueous phase. In the present case, the incorporation of the ammonium-selective ionophore favors the extraction of ammonium against possible competing cations, thereby adding selectivity to the measurement. The peak potential associated with the ion transfer process is governed by the sum of the redox potential of the redox probe, *E*_Redox_, and the phase-boundary potential of the transferred ammonium, $\Delta_{aq}^{m}\Phi_{j^{+}}$ .^19^ An ammonium concentration change alters the phase-boundary potential and consequently modulates the potential at which reduction of the TEMPO occurs, which is the fundamental basis for the imaging principle.

The optical response arises directly from electrochemical transformation of the redox probe.^6^ Rhodamine dyes were reported to undergo fluorescent quenching in the presence of TEMPO radicals.^20,21^ Consequently, progressive electrochemical reduction of cationic species of lip-TEMPO progressively increases the concentration of the lip-TEMPO radical, leading to a concomitant quenching of the dye fluorescence. Consequently, the potential at which maximum fluorescence decrease is observed for each pixel provides a direct optical readout of the electrochemically driven ammonium transfer process at the membrane-solution interface.

### Introducing Selectivity

The strategy proposed to introduce selectivity into Voltammetric Ion Transfer Microscopy relies on the incorporation of a selective ionophore in the sensing membrane composition. To evaluate this concept, ammonium was selected as model analyte and selective recognition was introduced using a new ammonium selective ionophore recently characterized by our group.^13^ Owing to its tripodal architecture, it exhibits improved selectivity toward ammonium compared to its widely used alternative nonactin.^10,22^

The accessible concentration range for ammonium under the experimental conditions used throughout this work was evaluated by performing a calibration from 1 μM to 100 mM (see Figure S4). The lower limit of detection is determined by the supporting electrolyte and was estimated using the Nikolsky equation (see equation S9) to be 21 μM. At higher concentrations, the response remains linear up to 100 mM and follows Nernstian behavior with a slope of 59.19 mV.

To assess the performance of the ionophore within the VITM imaging platform, a series of 10 mM nitrate solutions of different possible interfering cations with 25 mM MgCl_2_ as background electrolyte were tested. Besides NH_4_^+^, the investigated possible interferent cations included Ca^2+^, Li^+^, Na^+^ and K^+^. The recorded voltammograms are shown in Figure 2a, while the corresponding optical intensity difference curves are given in Figure 2b. The corresponding peak potential maps for the entire electrode surface obtained after image processing are presented in Figure 3.

**Figure 2:**
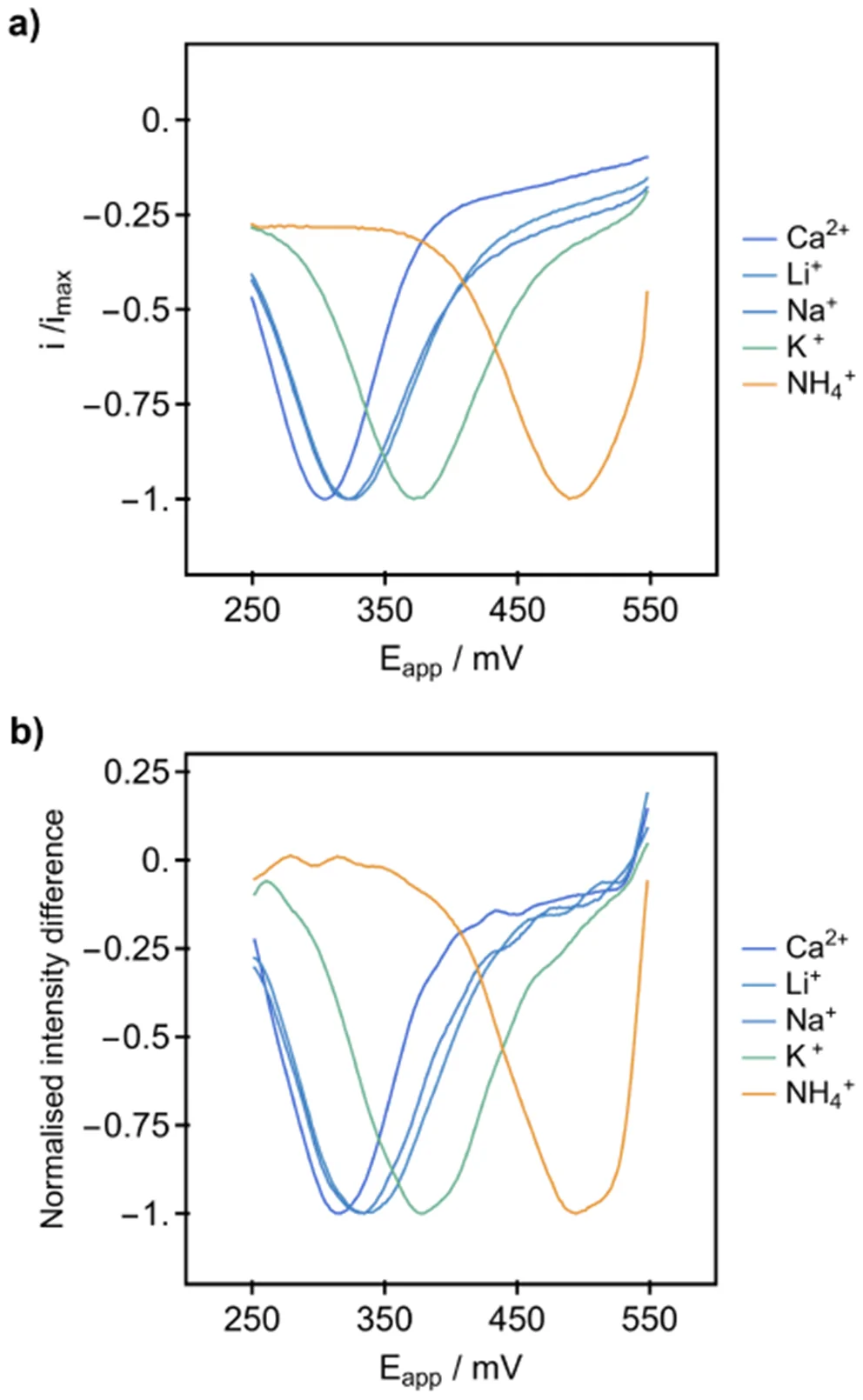
Determination of the electrochemical and optical selectivity coefficients of the ammonium selective membrane for ammonium over different interfering ions. (a) Normalized cathodic linear potential scans recorded from 550 to 250 mV (vs Ag/AgCl, 15 mV s^–1^) in solutions containing 10 mM of the nitrate salt of the ion of interest in a background of 25 mM MgCl_2_. (b) Simultaneously acquired optical response from intensity differences of consecutive image frames. The data is then expressed as peak potential for each pixel, which is visualized as an image (Figure 3). Fluorescence intensities were normalized by the maximum peak intensity.

**Figure 3:**
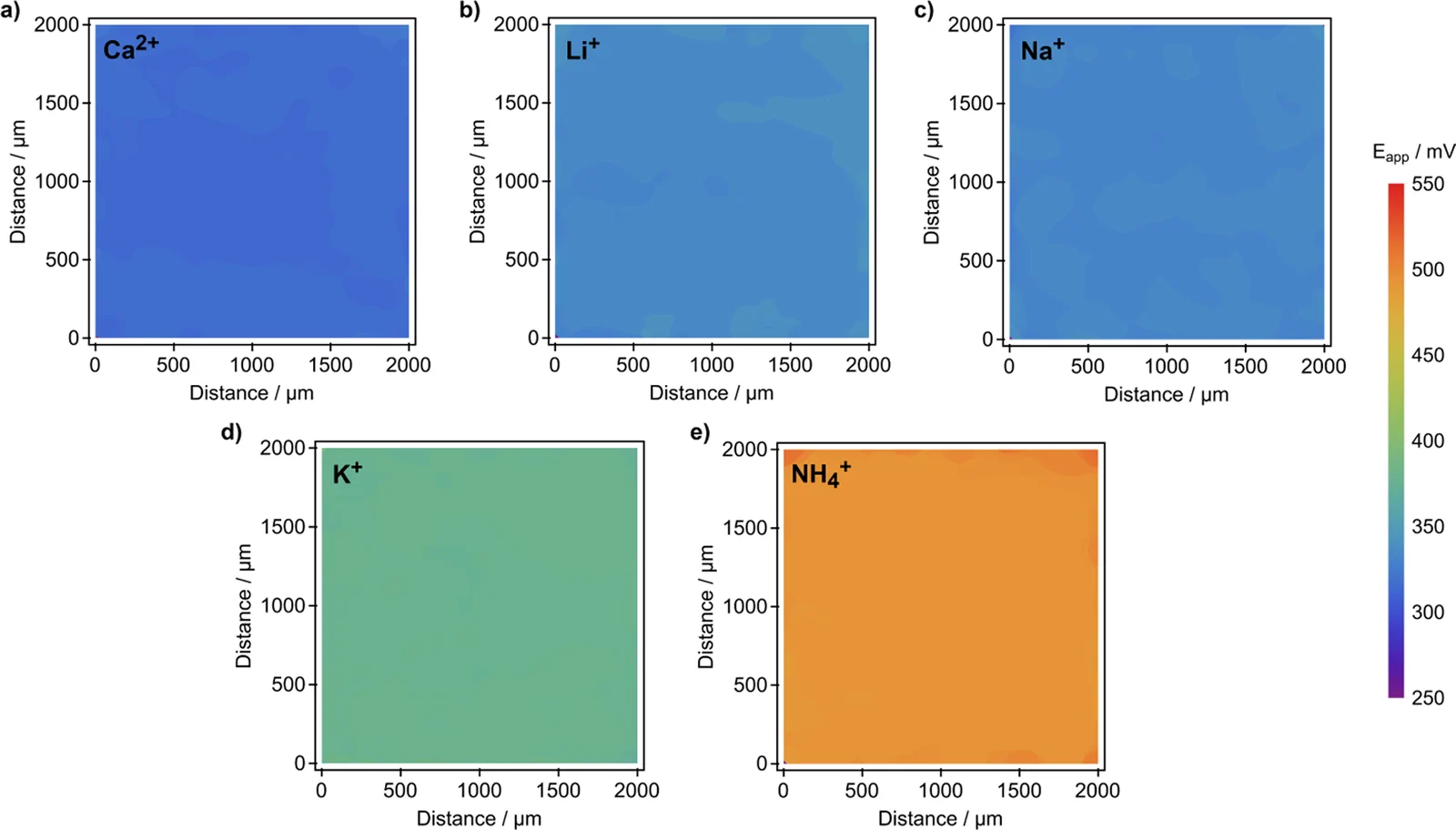
Density plots of the transition potentials (*E*_Peak_) extracted for each pixel in the image stack (visualized with a false-color scale) for the determination of the optical selectivity coefficients of the ammonium selective ionophore. Measurements were performed in 10 mM nitrate salt solutions of the following ions: (a) Ca^2+^, (b) Li^+^, (c) Na^+^, (d) K^+^, and (e) NH_4_^+^ in a background of 25 mM MgCl_2_. The peak potentials and associated standard deviations are given in Table S1.

For the optical measurements, the fluorescence intensity change of every pixel in the image was extracted throughout the full image sequence and the average response was calculated to generate the curves given in Figure 2b. The corresponding electrochemical and optical peak potentials for each cation are summarized in Table S1.

A comparison of Figures 2a and 2b shows a good agreement between the electrochemical and optical data to within 0.1 logarithmic units, which demonstrates that the optical responses in the selective system faithfully reproduce the electrochemical measurements and that the incorporation of the ionophore does not alter the fundamental coupling between the electrochemically driven ion transfer and the fluorescence modulation. We note that the optically derived curves exhibit a higher noise than the electrochemical data. This originates from the relatively small fluorescence modulation (approximately 20% change) associated with the conversion of lip-TEMPO to its radical form, which limits the signal-to-noise ratio. The fluorescence modulation obtained during the cathodic scan was lower than previously reported for the anodic measurements, which is consistent with the lower total charge transferred during the cathodic scan. Additionally, the fluorescence change being expressed relative to the initial fluorescence intensity, the higher initial fluorescence before the quenching by the radical form of lip-TEMPO contributes to smaller relative intensity changes. This difference does not appear to be associated with the incorporation of the ionophore and would benefit from further investigation. Additionally, slight differences in peak width and shape are visible and can eventually be explained by the nonlinear response of the dye in the presence of lip-TEMPO as reported previously.^6^ As a consequence, small variations in the measured peak potentials of up to 10 mV are observed when comparing the two techniques (see Table S1).

Despite these small deviations, the optical selectivity coefficients reproduce the electrochemically determined selectivity sequence and is consistent with that previously reported for this ionophore (Table 1).^13^

**Table 1:** Electrochemical (EC) and Optical (Opt) Selectivity Coefficients for Ammonium over Different Interfering Ions Determined by VITM^a^

Interfering ion	${\log\left(K_{i,j}^{pot}\right)}_{EC}$	${\log\left(K_{i,j}^{pot}\right)}_{Opt}$
Ca^2+^	–4.16 ± 0.01	–4.03 ± 0.04
Li^+^	–2.84 ± 0.02	–2.70 ± 0.04
Na^+^	–2.89 ± 0.01	–2.74 ± 0.04
K^+^	–2.01 ± 0.02	–1.96 ± 0.04

a The corresponding potential peaks are given in Table S1.

The chemical maps in Figure 3 give well-behaved spatial heterogeneity. The observed peak potential variations vary on the order of 1 mV (see Table S1), which may originate from uncertainties in the peak determination using the automated image analysis routine that are limited by the fluorescence signal change and associated noise. Overall, this demonstrates that the optical imaging provides quantitative selectivity information consistent with the conventional electrochemical measurements.

### Visualizing Ammonium Production by Enzymatic Activity in Microfluidic Flow

To further illustrate the proposed strategy for introducing chemical selectivity into VITM, the methodology was evaluated by monitoring ammonium produced during an enzymatic reaction. jack bean urease (*Canavalia ensiformis*) was selected as a model system because it catalyzes the hydrolysis of nonredox active urea into ammonia without generating any electroactive byproducts. Consequently, any observed signal change should originate exclusively from the enzymatically produced ammonium that is detected by the ion-selective membrane. This makes this reaction particularly suited to demonstrate the capabilities of the proposed approach. The corresponding reaction is the following:

$$Urea+2H_{2}O\overset{Urease}{\to }{H_{2}CO}_{3}+{2NH}_{3}$$

Under the experimental conditions employed here (30 mM MOPS buffer, pH 7.40), the ammonia produced by the reaction is predominantly protonated to ammonium (p*K*_a_(NH_4_^+^/NH_3_) ≈ 9.25 at 25 °C).^23^ This pH was selected as it corresponds to the optimal working condition of jack bean urease.^24^ In addition, the pH response of the ionophore has been previously investigated and shown to have no significant interference under these experimental conditions.^13^

To quantitatively predict the ammonium concentration profile expected during VITM measurements, the kinetic parameters of the urease-catalyzed reaction were determined under the same experimental conditions used throughout the work, as enzyme activity depends strongly on temperature and pH.^25^ Reaction kinetics were characterized potentiometrically with an ammonium-selective electrode (see Supporting Information for details) at laboratory ambient temperature, corresponding to the operating conditions of the microscope. The kinetic parameters were determined as *K*_m_ = 1.43 mM and *k*_cat_ = 5851 s^–1^, values that agree well with those reported in the literature.^24–27^ The data were subsequentially used in the mathematical predictions (for details see Supporting Information).

To exploit the spatially resolved capabilities of VITM, a well-defined ammonium concentration distribution was needed. For this, measurements were performed using a previously reported opto-electrochemical flow cell. A photograph and schematic representations are shown in the SI (Figure S8).

The expected ammonium concentration profile during the VITM experiment in the flow cell was estimated before performing the measurements. The local ammonium production was calculated based on Michaelis–Menten kinetics, which was then combined with diffusional mass transport across the flow junction. The time dimension of the simulation was then converted to distance by the linear flow rate of the experiment.

These simulations were used to identify the flow rate and the initial enzyme and substrate concentrations that would be expected to produce an ammonium concentration profile in the flow cell that could be resolved by VITM. The flow rate determines the residence time of the reaction within the observation window and therefore the time for enzymatic production and diffusive broadening. An excessive flow rate provides insufficient reaction time, resulting in a weak and spatially narrow concentration gradient in addition to the risk of delaminating the membrane by shear force.^28^ Too low flow rates produce a broad concentration profile with reduced spatial resolution. An intermediate flow velocity of 0.2 mm s^–1^ was therefore chosen to generate a well-defined concentration gradient over the imaged region spanning 2 mm along the flow direction, corresponding to 10 s of reaction time. A urease concentration of 100 nM was chosen to generate sufficient ammonium within the available residence time while keeping the expected concentrations within the calibration range of the ammonium selective membrane. A high urea concentration of 100 mM allowed us to maintain substrate-saturated conditions, giving a reaction rate close to *v*_max_ that allows the ammonium production rate to be considered constant over the observation time. A schematic representation of the experimental configuration is shown in Figure 4 and the resulting predicted ammonium concentration profile is presented in Figure 6b and 6d.

**Figure 4:**
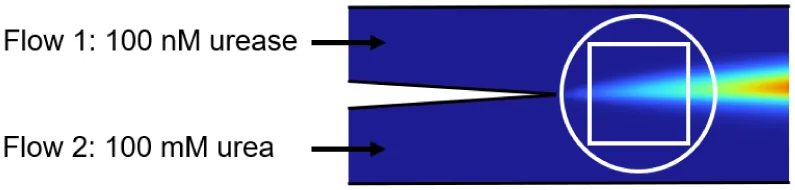
Schematic representation of the experiment performed in the opto-electrochemical flow cell (Figure S8) to visualize the spatial distribution of NH_4_^+^ produced by jack bean urease. Solutions containing either 100 nM urease (flow 1, top) and 100 mM urea (background electrolyte: 25 mM MgCl_2_; flow 2, bottom) were introduced in the cell. Upon contact, enzymatic hydrolysis generates NH_4_^+^. The simulation shows the expected spatial concentration distribution.

The simulation predicts the progressive formation of ammonium concentration originating at the interface between the enzyme and substrate solutions. As the solutions are transported downstream, the concentration profile broadens. The resulting concentration distribution is not perfectly symmetrical, which is explained by the significantly lower diffusion coefficient of urease compared to that of urea and ammonium.^23,29,30^ While urea diffuses rapidly across the interface into the enzyme stream, urease remains largely confined to its side during the considered reaction window. Consequently, the effective reaction zone is predicted to shift toward the enzyme side, leading to an asymmetry in the concentration profile.

Prior to the enzymatic experiments, the flow cell was calibrated using defined ammonium concentrations. The experimental procedure follows the protocol reported previously.^6^ The resulting transition potential density plot (Figure S11) resolved the two flowing solutions of different ammonium concentrations with an interfacial gradient. As expected, the width of this interfacial region progressively increased along the flow direction. This reflects lateral diffusion of ammonium during transport through the channel. These results are in good agreement with the simulation realized in Wolfram Mathematica (Figure S11b). The tridimensional representation of the experimental data in Figure S11c visualizes the potential step between the two solutions. The experimentally measured potential difference of 117.5 mV is close to the predicted response of 118.3 mV (Figure S11d) expected for 100-fold concentration change. It confirms that the membrane follows Nernstian behavior.

The VITM experiment was subsequentially implemented with the conditions defined by the simulation. Two solutions containing 100 nM urease and 100 mM urea, respectively, were introduced into the opto-electrochemical flow cell (Figure 4). Within the inlet region, the two solutions are initially separated by a physical barrier. When reaching the ISM, the separation ends, and the channel geometry allows the two solutions to continue flowing in parallel with a contact surface at their interface allowing for diffusion perpendicular to the flow direction and so the begin of the enzymatic reaction. Ammonium is thus generated in situ at the interface and then transported downstream by convection while simultaneously spreading laterally by diffusion.

The VITM protocol was applied under continuous flow conditions. The recorded voltammogram reveals a stable and reproducible ammonium signal confirming a steady-state production regime (Figure 5). This stationary regime is the consequence of the continuous renewing of reactants, which establishes a constant spatial distribution of reaction products within the sensing region. While the electrochemical measurement alone confirms the enzymatic generation of ammonium, it does not give any spatial information on its distribution.

**Figure 5:**
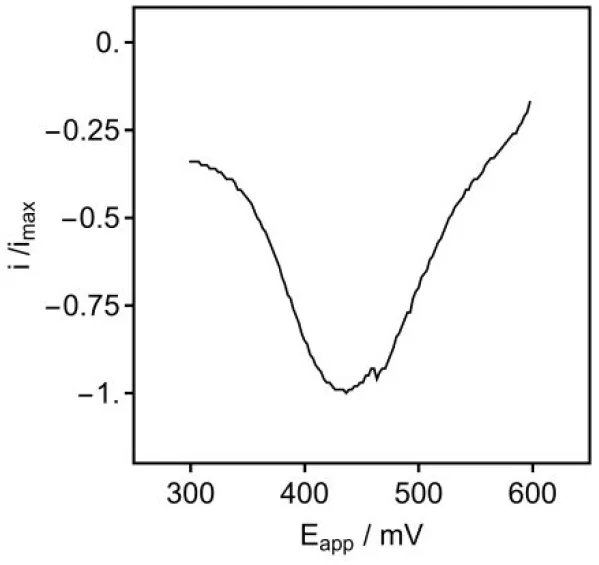
Normalized linear potential scan recorded from 550 to 250 mV (vs Ag/AgCl, 15 mV s^–1^) of NH_4_^+^ production by enzymatic activity in the flow cell (Figure S8) upon contact between the two flowing solutions (respectively 100 nM jack bean urease and 100 mM urea, both in 25 mM MgCl_2_ background).

In contrast, the simultaneously acquired optical measurements (Figure 6a and 6c) allow for the spatial visualization of ammonium concentration across the channel. The peak potentials determined with the automated image analysis routine were converted to ammonium concentrations using the previously established calibration (see intermediate potential density plots and corresponding tridimensional representations in Figure S12). The spatial resolution of this VITM setup was previously established to be one pixel, corresponding to 1.3 μm with the 5x objective used here.^6^ Consequently, the concentration profile is reconstructed at discrete spatial positions, resulting in stepwise appearance. For the present concentration map, the smallest observed concentration step is approximately 3.0 μM. The experimentally reconstructed concentration map (Figure 6a and 6c) can be directly compared with the simulated distribution (Figure 6b and 6d).

**Figure 6:**
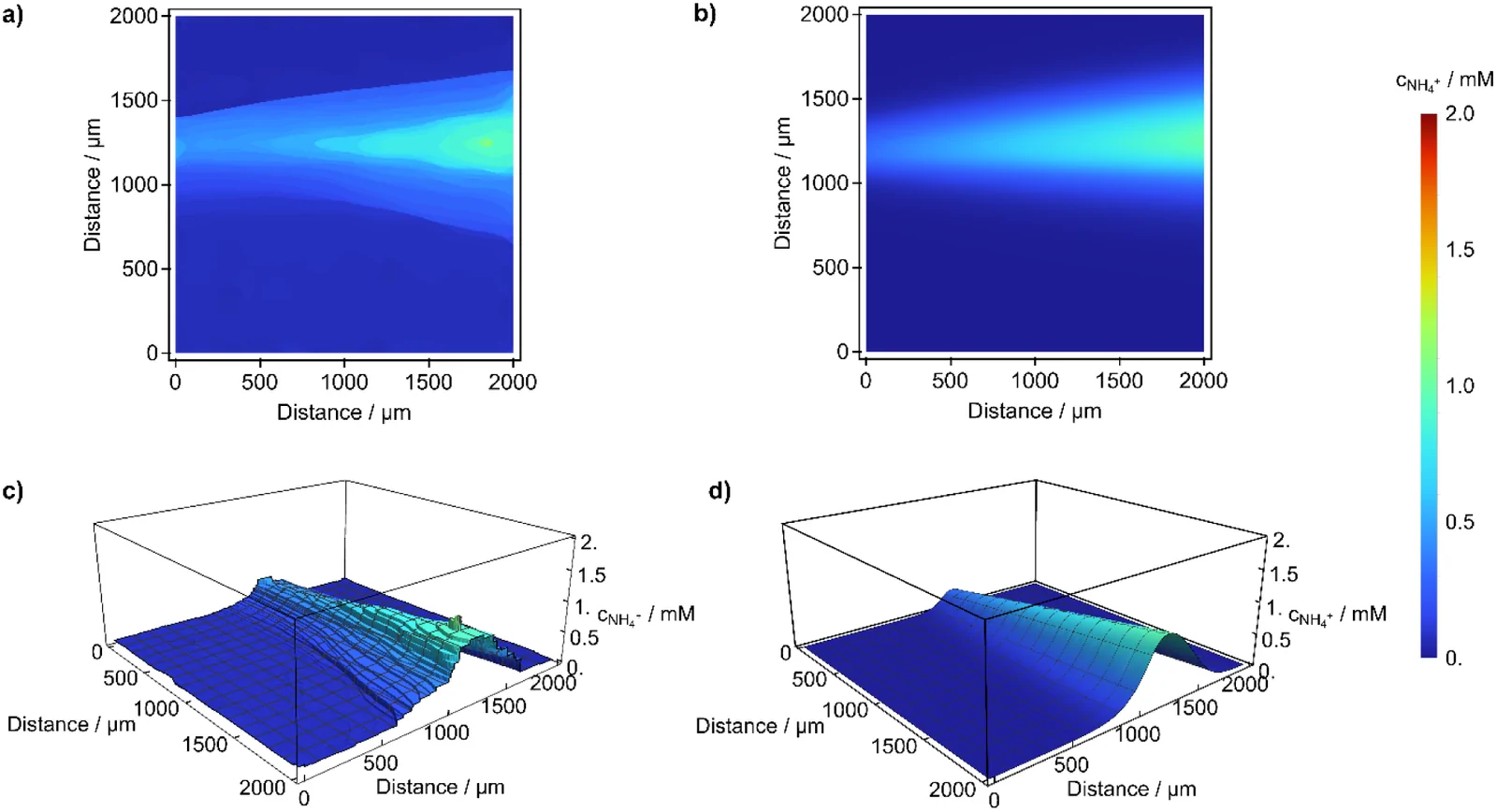
Experimental and simulated concentration distribution of NH_4_^+^ produced by jack bean urease, while flowing two solutions containing, respectively, 100 nM of the enzyme and 100 mM of urea (background electrolyte: 25 mM MgCl_2_) in the opto-electrochemical flow cell. (a) Concentration map recorded by the ion-selective membrane. (b) Corresponding simulated ammonium concentration distribution. (c) and (d) show 3D representations of (a) and (b) respectively.

As predicted, ammonium first appears as a narrow low concentration band located at the interface between the enzyme and substrate flows. Along the flow direction the profile progressively broadens owing to the combined effect of continuous enzymatic production and lateral diffusion. The measured concentration gradient closely reproduces the predicted profile, both in terms of the spatial evolution and concentration level. In particular, the slight displacement of the concentration gradient toward the enzyme side of the membrane agrees well with the asymmetry predicted from the difference in diffusion coefficients.

The close agreement between experimental and simulated concentration profiles further confirms the ability of ionophore-based VITM to reliably and selectively resolve spatial ion distributions.

## Conclusions

This work presents the first ionophore-based implementation of Voltammetric Ion Transfer Microscopy, demonstrating a general strategy to introduce ion selectivity while preserving the fundamental opto-electrochemical sensing mechanism. While the original methodology was governed by the Hofmeister selectivity sequence, we demonstrate that incorporating an ionophore into the membrane enables the selective detection of a target ion beyond these intrinsic limitations. Here, ammonium was chosen as a model analyte to validate the concept.

For the first time, electrochemical and optical selectivity measurements were performed simultaneously with VITM. The resulting electrochemical and optical selectivity coefficients were found to be very close, with only a slightly lower selectivity observed optically. Improving the optical signal change should further enhance the signal-to-noise ratio, thereby improving both the precision in peak potential determination and so the quality of the reconstructed chemical maps.

As a proof of concept, the method was successfully applied to monitor the spatial distribution of ammonium produced by enzymatic activity of jack bean urease in a microfluidic system. The excellent agreement between experimental observations and simulations further validates the proposed approach.

Because this strategy relies solely on the incorporation of an appropriate ionophore, it can, in principle, be readily extended to other target ions for which suitable ionophores are available. Ionophore-based VITM therefore provides a versatile platform for selective ion imaging and opens new opportunities for spatially resolved measurements in chemically more complex systems in analytical, bioanalytical and environmental applications. Future developments could for example extend its applicability to extracellular imaging of ionic gradients in biological samples, where spatially resolved measurements of nonredox active ions remain challenging. Such applications would require further optimization of the membrane and imaging configuration, including a transparent working electrode to facilitate optical access to biological samples.

## Supplementary Material



## Data Availability

The original data and calculation files for this publication are available at https://doi.org/10/hcfhxx.

## Notes

Determination of enzymatic parameters, development of simulations, and supplementary photographs, figures, schemes, and tables for selectivity measurements, calibration, and ammonium production in the flow cell (PDF)
