# Supplementary material for: Ionophore-Based Voltammetric Ion Transfer Microscopy: Imaging Enzyme-Generated Ammonium in Flow
Source: ACS Meas Sci Au. 2026 Sep 11;6(5):1866–74. doi: 10.1021/acsmeasuresciau.6c00226 (PMC13693184; doi:10.1021/acsmeasuresciau.6c00226)
Supplement: Supplementary file 1 [file tg6c00226_si_001.pdf]

Supporting Information for:

Ionophore-Based Voltammetric Ion Transfer Microscopy: Imaging Enzyme-Generated Ammonium in Flow

Justine A. Rothen, and Eric Bakker*

Department of Inorganic and Analytical Chemistry, University of Geneva, 1211 Geneva, Switzerland

*corresponding author: eric.bakker@unige.ch

1. Determination of Michaelis-Menten kinetic parameters of Jack bean urease by potentiometry

Preceding measurement of ammonium production in the flow cell, kinetic parameters and enzymatic activity of the Jack bean urease were determined in the experimental conditions (20°C, pH 7.40) by potentiometric measurements.

Electrode preparation

First, glassy carbon working electrodes were polished sequentially with diamond powder suspensions of decreasing particle size (Ø 6, 3, 1 and 0.25 µm).

A tape mask with a circular opening (5.5 mm diameter) was placed on the electrode surface to define the transducing layer deposition area. Three successive 15 µL aliquots of a carbon nanotubes suspension (1mg in 1mL THF) were drop-cast on the electrode surface at 20 min intervals to allow for the solvent to evaporate. After being fully dry, the masking tape was gently removed.

The ion-selective membrane cocktail subsequently deposited consists of equimolar concentrations of redox probe (lip-TEMPO), cation-exchanger (NaTFPB) and ammonium-selective ionophore (50 mmol kg^{-1}) and 15 mmol kg^{-1} of ETH 500. The polymeric matrix is based on PVC and the plasticizer NPOE at a mass ratio of 1:2 (w/w). The total mass added in the 1 mL THF is 50 mg.

In a similar manner, three consecutive 50 µL aliquots of the membrane cocktail were drop-cast at intervals of 20 min. The membrane was then left to dry overnight.

Finally, the electrodes were conditioned overnight in 1 mM NH_4NO_3 (in 30 mM MOPS buffer adjusted to pH 7.40).

Potentiometric measurements

All measurements were realised in 30 mM MOPS adjusted to pH 7.40.

A potentiometric calibration was first performed in NH_4NO_3 solutions with concentrations ranging from 10^{-4} M to 10^{-2} M (Figure S5) in order to correlate the potentiometric response to the activity.

Then, the enzymatic activity of Jack bean urease (100 nM) was monitored by measuring the potential generated by ammonium production resulting from enzymatic hydrolysis of urea. The measurement was repeated for the following initial substrate concentrations: 0.1, 0.3, 0.5, 1, 5, 10, and 30 mM.

Determination of the enzymatic parameters

The Michaelis-Menten plot was obtained by extracting the initial reaction rate v_0 (linear portion of the slope at the beginning of the reaction, expressed in mV s^{-1}) for each substrate concentration. The Lineweaver-Burk representation was then used to determine the K_m and the v_{max}:

Lineweaver-Burk:

$$\frac{1}{v_0} = \frac{K_m}{v_{max}} \cdot \frac{1}{c_{urea}} + \frac{1}{v_{max}} \qquad \text{S1}$$

The values for K_m and v_{max} were then determined using the abscissa and ordinate intercepts, respectively:

$$\frac{1}{v_{max}} = \frac{K_m}{v_{max}} \cdot 0 + \frac{1}{v_{max}} \qquad \text{S2}$$

$$0 = \frac{K_m}{v_{max}} \cdot \frac{1}{c_{urea}} + \frac{1}{v_{max}} \qquad \text{S3}$$

The K_m was determined to be 1.43 mM, which is consistent with values commonly found in the literature.[1,2] Concerning the v_{max} under these experimental conditions, it was found to be 3.81 mV s^{-1}, corresponding to 0.29 µM s^{-1} after conversion using the calibration curve.

To prepare initial stock solutions of urease, the theoretial enzyme activity provided by the manufacturer (372 U mg^{-1}, determined at 25°C and pH 7.0) was used as an initial reference. However, as the experiments were performed under laboratory conditions (20-22°C) in a MOPS buffer adjusted to pH 7.40, the effective enzymatic activity was determined. It was estimated from the amount of NH_4^+ produced by the enzyme in the experimental conditions, giving an activity of 594.78 U mg^{-1}.

Finally, the catalytic constant was calculated using the following equation:

$$k_{cat} = \frac{v_{max}}{c_{urease}} \qquad \text{S4}$$

A value of 5850.7 s^{-1} was obtained for k_{cat}, which is in good agreement with previously determined values.[3]

The parameters obtained are alongside the experimental values for the simulations described in the next section.

2. Simulations

Simulations in Wolfram Mathematica were performed using the experimental parameters to predict the expected theoretical response in the flow cell and compare it with the experimental results to validate that the observed behaviour is consistent with the predictions.

The two experiments having been realised in the same flow cell, the following geometrical parameters were used for both simulations:

Distance parameters in the flow cell: xmin = 0 mm; xmax = 4.2 mm; xInterface = 2.1 mm; dx = 0.1 mm

Time parameters: tmemb = 10 s (time needed for the solutions to flow over the total distance of the membrane region selected for analyses with a velocity of 0.2 mm s^{-1}.)

Ammonium calibration in the flow cell

The parameters used for the simulations of the NH_4^+ calibration in the flow cell are the following:

Diffusion coefficient: $D_{NH_4^+}$ = 1.96 · 10^{-3} mm^2 s^{-1}.[4]

Initial conditions : $c_{NH_4^+,hcInit}$ = 10 mM (high initial concentration); $c_{NH_4^+,lcInit}$ = 0.1 mM (low initial concentration).

Boundary conditions: There are no fluxes flowing outside of the flow cell: $\left.\frac{dc}{dx}\right|_{x=0} = \left.\frac{dc}{dx}\right|_{x=xmax} = 0$.

To simplify the model, the simulation was done stepwise and the diffusion process of NH_4^+ was first modeled in one dimension, which corresponds to a single-cross-sectional profile perpendicular to the flowing direction (Figure S1). This simplification is justified because the concentration profile at different positions along the flow cell corresponds to the same diffusion process at one distance point, but observed at different times.

For the one-dimensional diffusion of ammonium, Fick's second law of diffusion was applied:

$$\frac{dc(x,t)}{dt} = D\frac{d^2(x,t)}{dx^2} \qquad \text{S5}$$

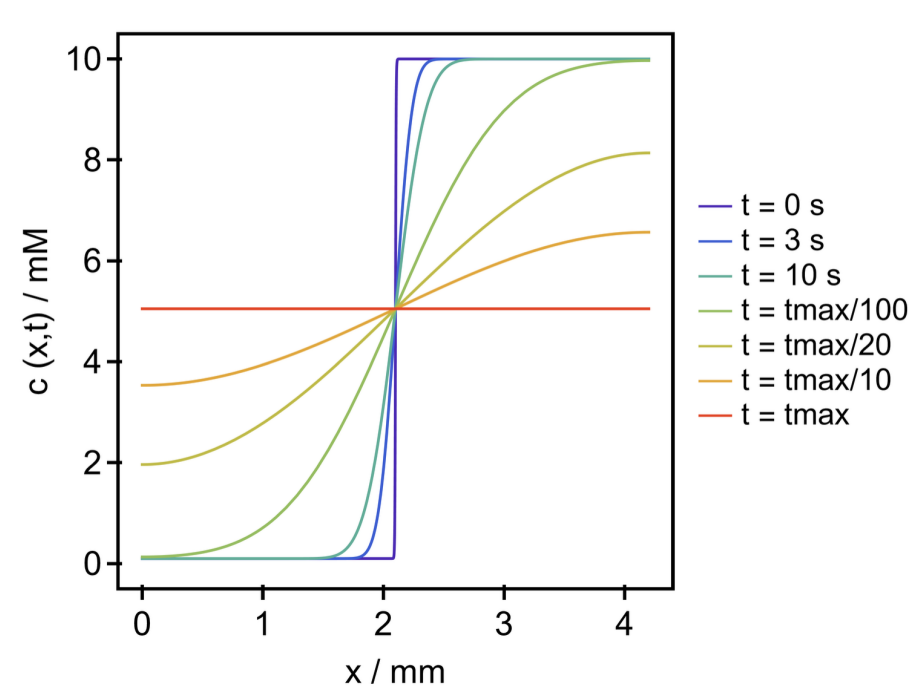

Figure S1. 1D simulation of ammonium diffusion between two initially separated solutions of different NH_4^+ concentrations, 10^{-4} M (0-2.1 mm) and 10^{-2} M (2.1-4.2 mm), in the flow cell (concentration profile perpendicular to the solution flow). At t_0, the separation is removed, allowing diffusion between the two solutions to occur. The concentration profiles are shown at different time points until homogeneity is achieved.

After obtaining the concentration profiles for any time point, the second dimension was obtained by adding each concentration profiles at a given time point to its corresponding diffusion position in the flow cell according to the flow velocity (0.2 mm s^{-1}). This allowed to generate a bidimensional concentration profile, where the concentration is now visualised using a false colour scale.

Ammonium production by enzymatic activity of Jack bean urease in the flow cell

The parameters used for the simulation of the concentration profile of ammonium resulting from enzymatic production in the flow cell are given thereafter:

Diffusion coefficients: $D_{NH_4^+}$ = 1.96 · 10^{-3} mm^2 s^{-1} ; D_{Urea} = 1.38 · 10^{-3} mm^2 s^{-1} ; D_{Urease} = 3.18 · 10^{-5} mm^2 s^{-1} .[4–6]

For simplification, it was considered that the enzyme would not move during the time of the measurements (20 s) as its diffusion coefficient is two orders of magnitude smaller compared to those of the substrate and the product.

Initial conditions : c_{Urea} = 100 mM; c_{Urease} = 100 nM.

Boundary conditions: There are no fluxes flowing outside of the flow cell: $\left.\frac{dc}{dx}\right|_{x=0} = \left.\frac{dc}{dx}\right|_{x=xmax} = 0$

Concerning the enzymatic kinetic parameters, those given in Section S1 were used: k_{cat} = 5850.7 s^{-1}; K_m = 1.43 mM.

To develop this simulation, the same strategy as for the previously described simulation for the calibration in the flow cell was adopted. To simplify the development, the one-dimensional concentration profile of NH_4^+ production was first implemented (a potntio S2), then all profiles were added at their respective time points (distance in the flow cell) to generate the two-dimensional concentration distribution of ammonium produced by the enzyme with a false-colour scale representing the concentration.

Again, Fick's second law of diffusion (Equation S5) was applied to model the diffusion of urea, to begin with, and then that of ammonium when it got produced.

To describe the enzyme kinetics, the Michaelis-Menten equation was employed:

$$v = \frac{d[NH_4^+]}{dt} = \frac{k_{cat}[Urease][Urea]}{K_m + [Urea]} \qquad \text{S6}$$

Following this and according to the stoechiometry of the enzymatic reaction, the progressive consumption of urea and production of ammonium by urease can be expressed as follows:

$$\frac{dc_{Urea}(x,t)}{dt} = D_{Urea}\frac{d^2c_{Urea}(x,t)}{dx^2} - v \qquad \text{S7}$$

and

$$\frac{dc_{NH_4^+}(x,t)}{dt} = D_{NH_4^+}\frac{d^2c_{NH_4^+}(x,t)}{dx^2} + 2v \qquad \text{S8}$$

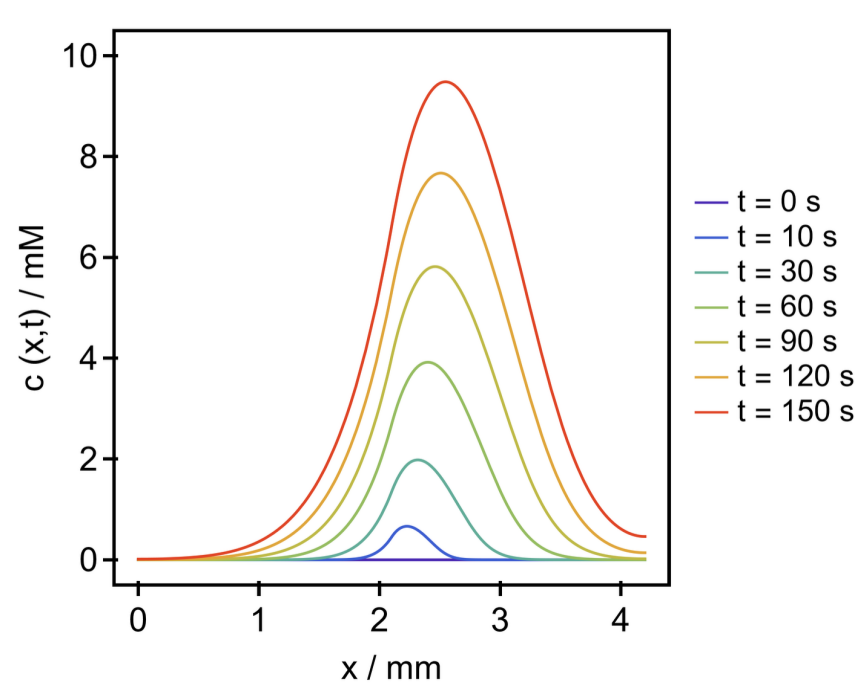

Figure S2. 1D simulation of the evolution of the ammonium concentration profile in time perpendicular to the solution flow in the flow cell. Initially, two solutions containing respectively 100 mM urea (0-2.1 mm) and 100 nM Jack bean urease (2.1-4.2 mm) are separated by a wall. At t_0, the separation is removed allowing for the two solutions to mix by diffusion, which initiates the enzymatic hydrolysis of urea and the production of NH_4^+. The different curves show the concentration profile at the indicated time points.

3. Supplementary figures, tables and images

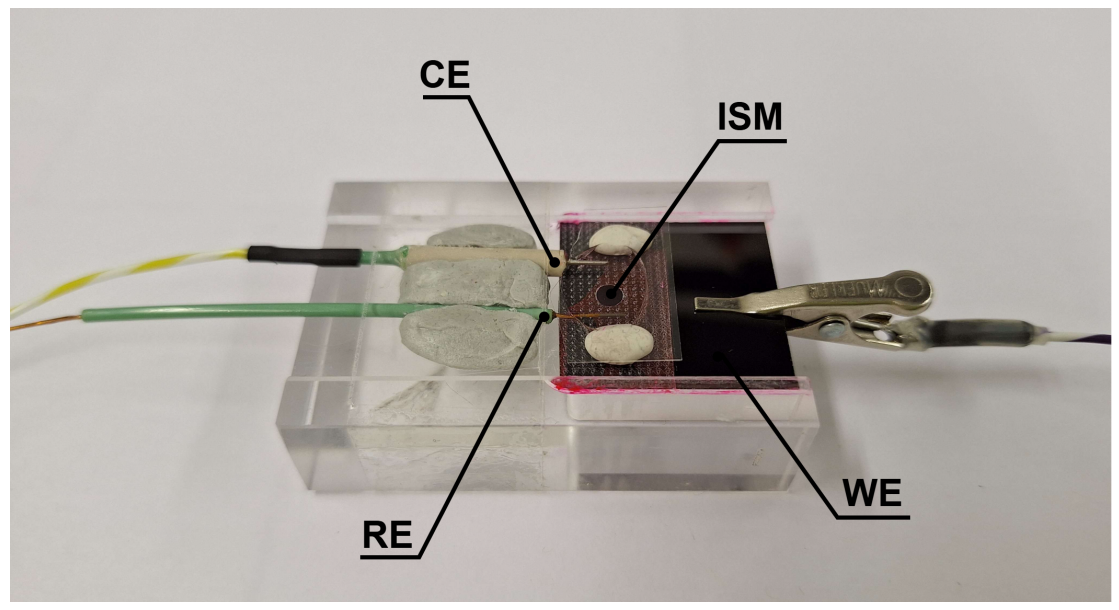

Figure S3. Photograph of the in-house built regular opto-electrochemical cell. The plexiglas base serves as support for the three-electrode configuration. The working electrode (WE, square glassy carbon plate) is inserted in a slit on one side of the base, while the quasi-reference electrode (RE, Ag/AgCl wire) and the counter electrode (CE, Pt wire) are mounted on a small plateform on the opposite side, which position them slightly above the WE. / This imaging platform was designed to be compatible with a commercial widefield fluorescence microscope.

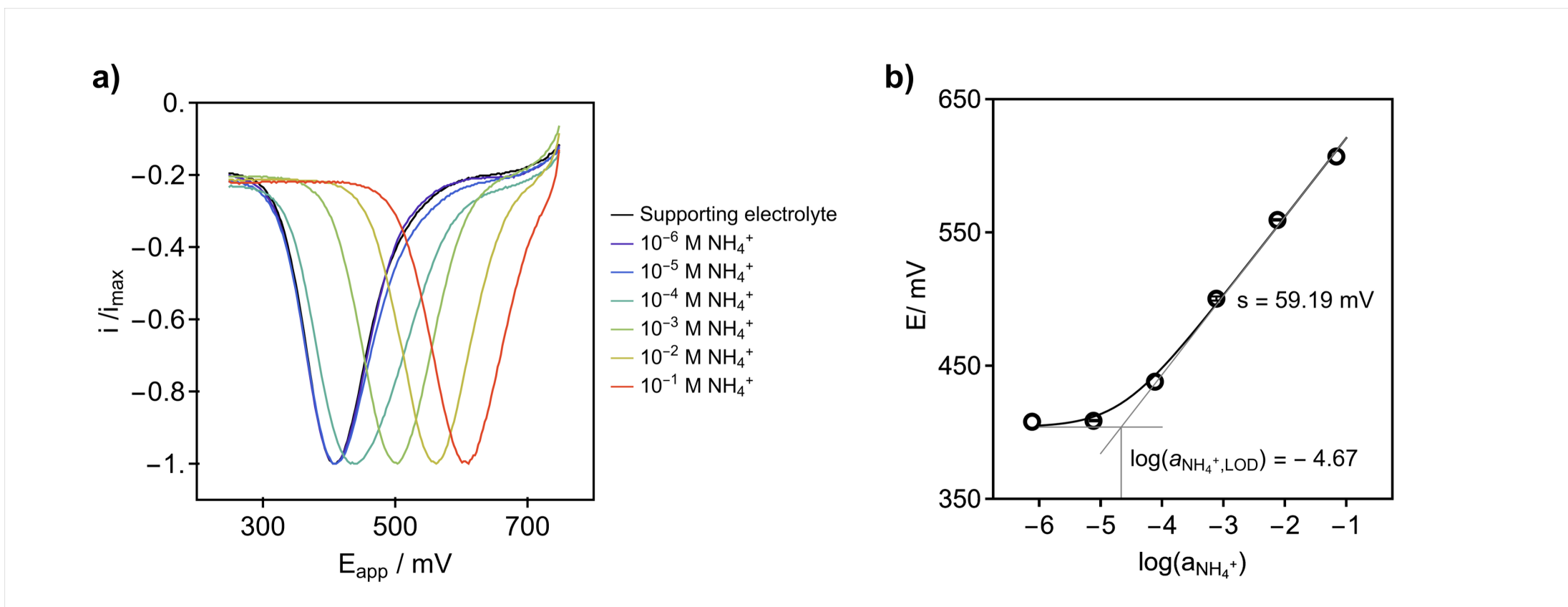

Figure S4. Voltammetric ammonium calibration. a) Normalized linear potential scan (15 mV s^{-1}) recorded in the supporting electrolyte alone (25 mM $MgCl_2$, black curve) and for solutions of increasing NH_4^+ concentrations (10^{-6} – 10^{-1} M) prepared in the same background. b) Calibration curve fitted using the Nikolsky equation. The lower limit of detection is emphasized at the intersection of the extrapolated linear regions.

Nikolsky equation:

$$E = E^0 + s \cdot log(a_i + K_{i,j}^{pot} \cdot a_j^{z_i/z_j}) \qquad \text{S9}$$

Table S1. Electrochemical ($E_{Peak,EC}$) and optical ($E_{Peak,Opt}$) peak potential values obtained from linear sweep voltammetry measurements for 10 mM nitrate salt solutions of the different ions in a 25 mM $MgCl_2$ background. The values for the mean and standard deviation from the electrochemical experiments were obtained from three replicates. For the optical values, the peak potentials determined for all pixels in an image were taken into account (2,250,000 points).

Interfering ion	$E_{Peak,EC}$ / mV	$E_{Peak,Opt}$ / mV
Ca^{2+}	304.7 ± 0.3	315.1 ± 1.4
Li^+	324.0 ± 1.0	335.1 ± 0.8
Na^+	321.3 ± 0.6	332.4 ± 1.1
K^+	373.0 ± 1.0	378.4 ± 0.3
NH_4^+	492.0 ± 0.0	494.7 ± 2.1

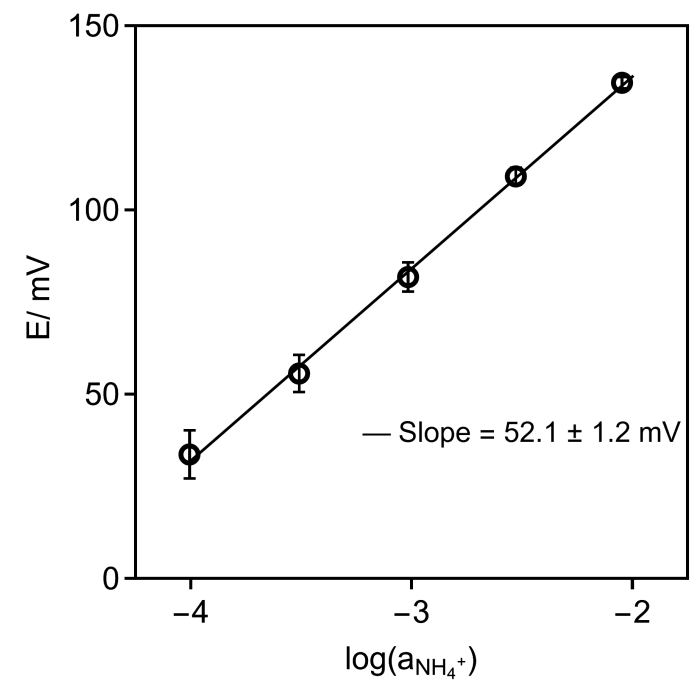

Figure S5. Potentiometric ammonium calibration curve.

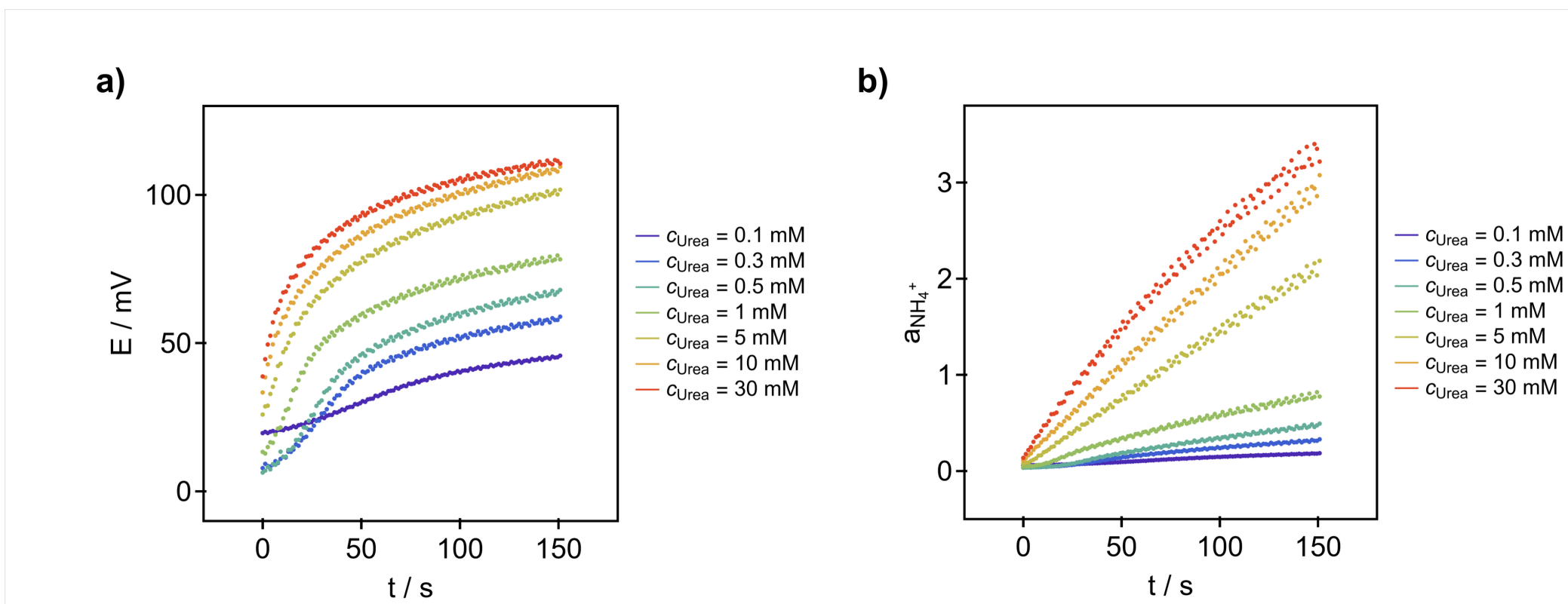

Figure S6. a) Potentiometric measurements showing the evolution of the potential corresponding to the enzymatic production of ammonium by Jack bean urease as a function of time for different initial concentrations of urea. b) Corresponding ammonium activity profile as a function of time.

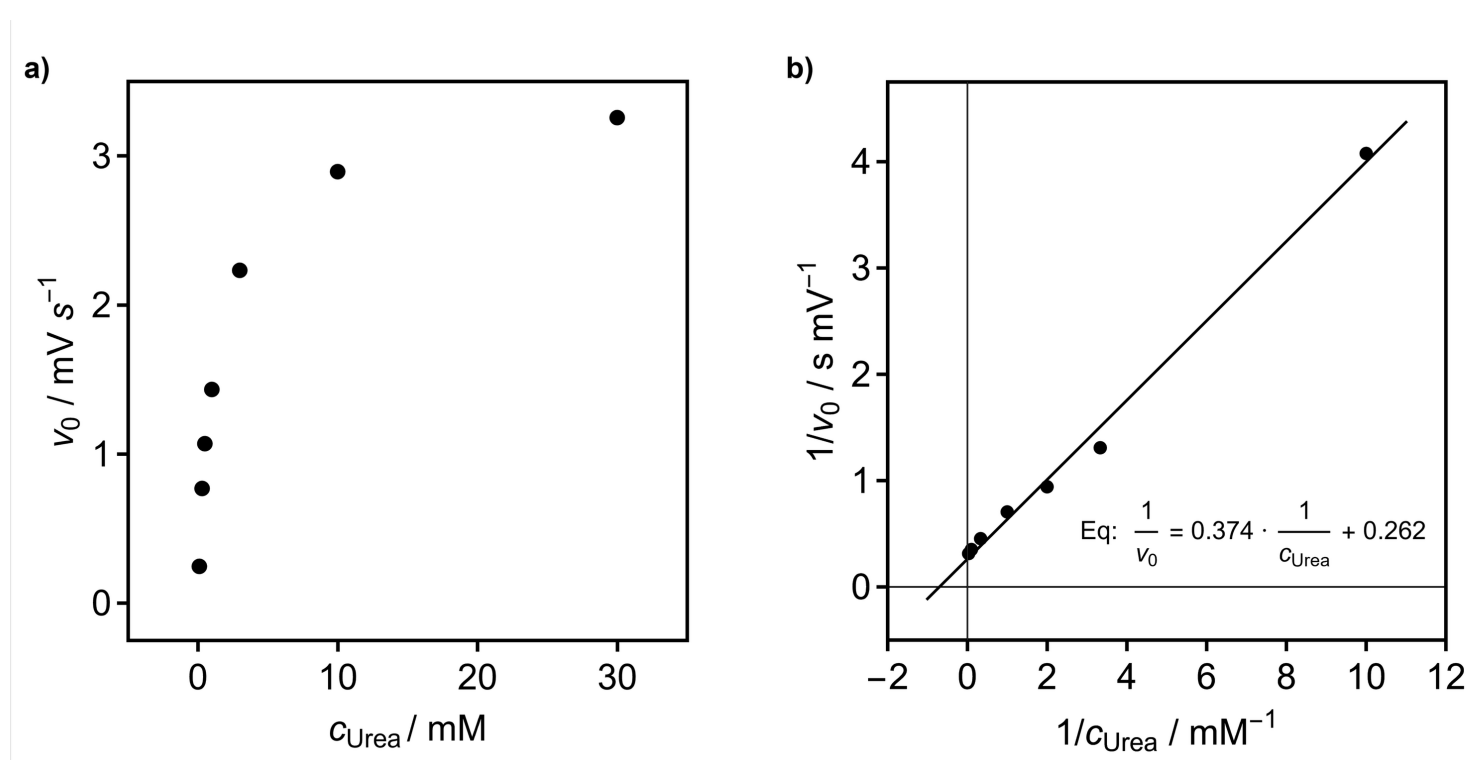

Figure S7. a) Michaelis-Menten plot showing the initial rate of ammonium production by Jack bean urease as a function of the initial concentration of urea. b) Corresponding Lineweaver-Burk representation.

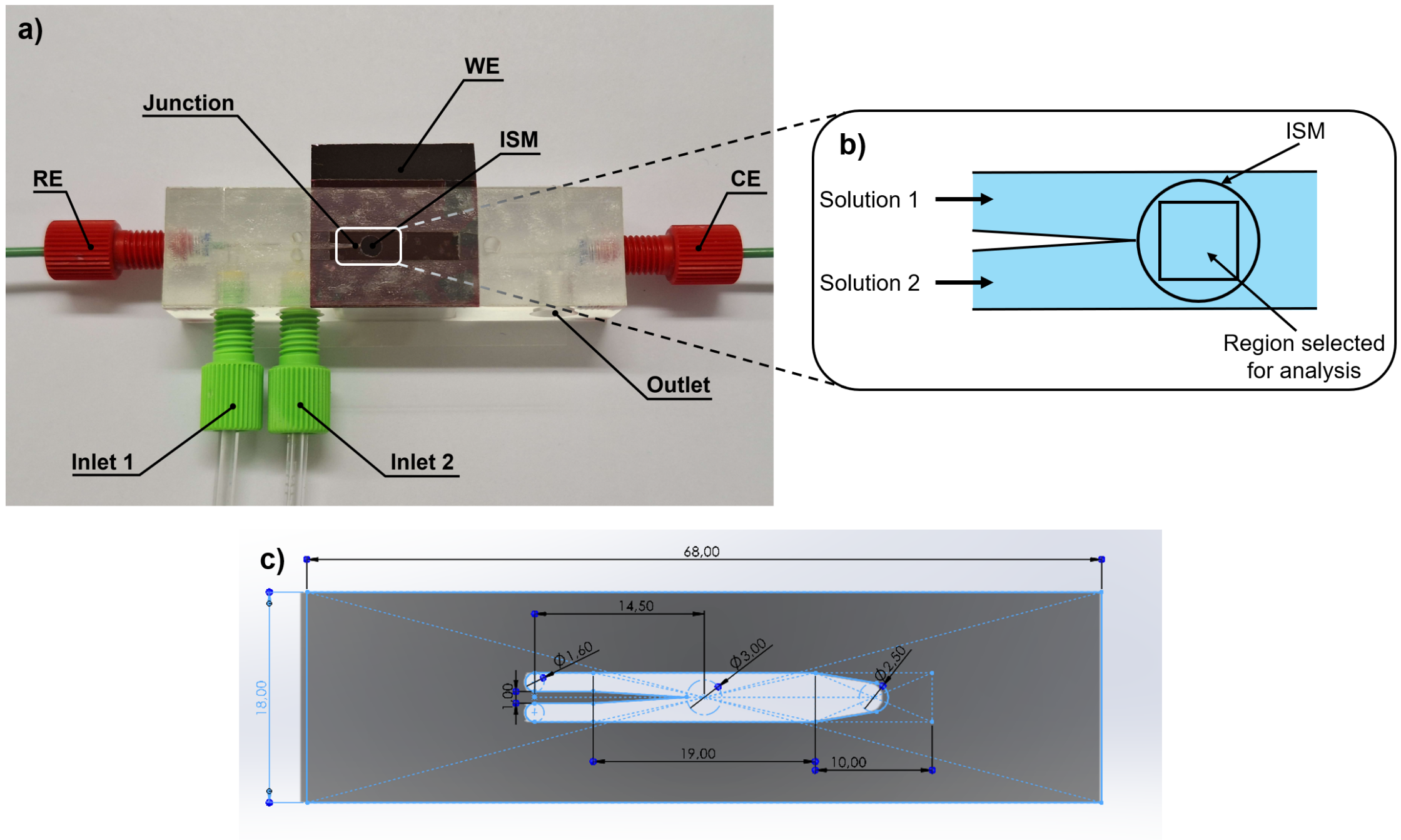

Figure S8. a) Photograph of the in-house designed and built opto-electrochemical flow cell. It is the same imaging platform that was first reported in a previous work of our group, where detailed schemes are provided.[7] This cell integrate a three-electrode configuration. Two inlets on the left allows for two solutions to flow in parallel over the sensing membrane (ISM). b) Schematic magnification of the region outlined in a). The two solutions are initially separated by a wall. This separation ends when reaching the ion-selective membrane (ISM, represented with the circle), allowing the two solutions to share a contact surface and so progressive mixing occurs. The square within the ISM shows the region selected for optical analysis. c) Dimensions of the flow cell channels.

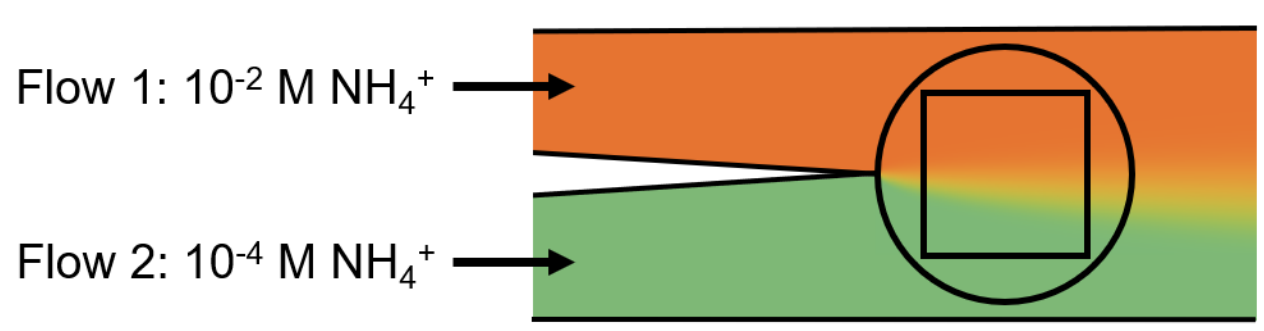

Figure S9. Schematic representation of the calibration performed in the flow cell. Two NH_4^+ solutions of different concentrations, respectively 10^{-2} M and 10^{-4} M (background electrolyte: 25 mM $MgCl_2$) were introduced in the cell. The simulation shows the progressive formation of a concentration gradient after the separating wall ends.

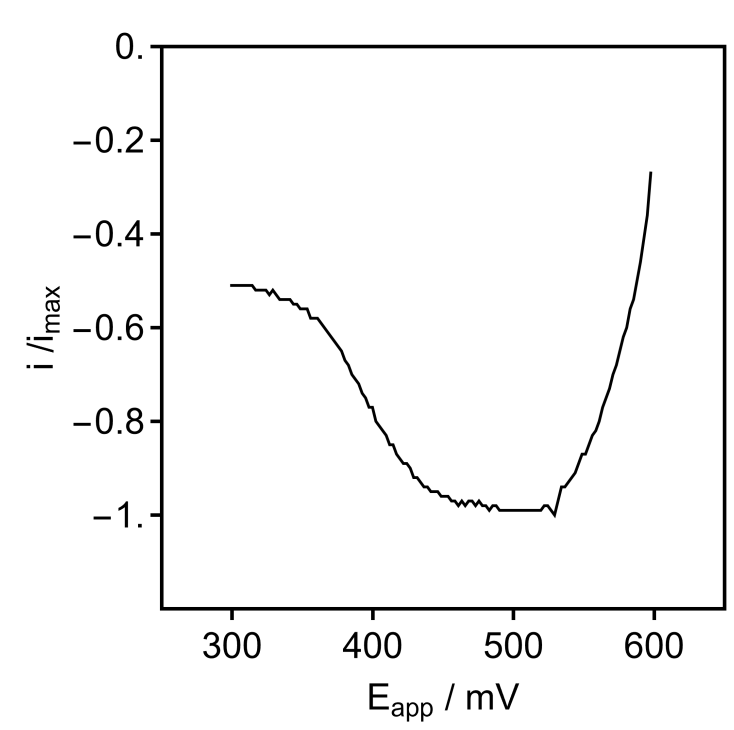

Figure S10. Linear potential scan (15 mV s^{-1}) acquired during the calibration in the flow cell where two NH_4^+ solutions of different concentrations (10^{-2} M and 10^{-4} M in a background of 25 mM $MgCl_2$) were flowed over the sensing membrane.

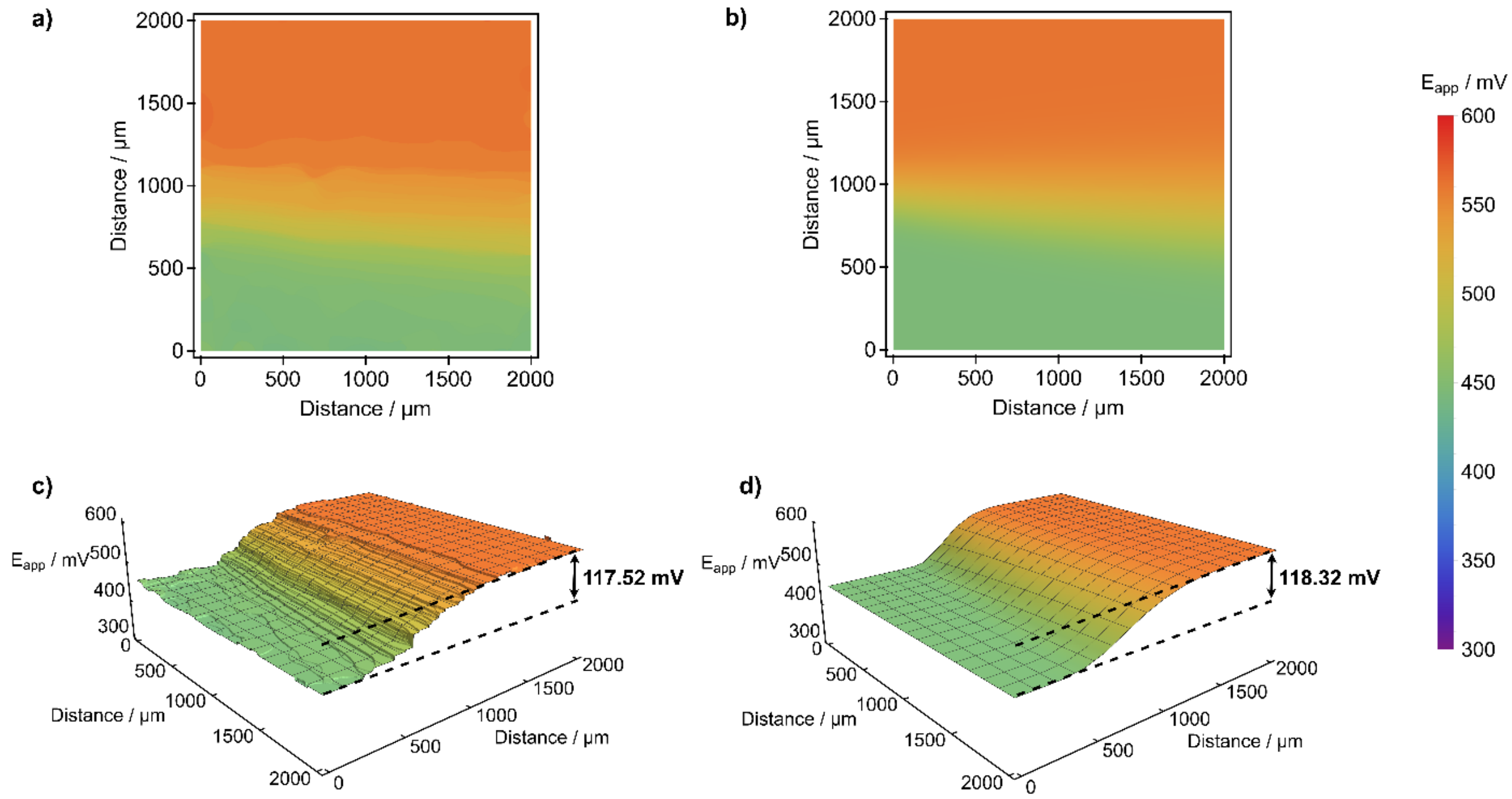

Figure S11. Experimental and simulated peak potential distribution obtained for the ammonium calibration in the flow cell while flowing two NH_4^+ solutions (10^{-2} M and 10^{-4} M in a background of 25 mM $MgCl_2$) over the sensing membrane. a) Density plot of the peak potentials measured for each pixel in the image. b) Corresponding simulated potential distribution. c) 3D representation of the experimental data shown in a). It highlights the potential step between the two solutions. The calculated potential difference is in agreement with a Nernstian behaviour. d) Simulated data corresponding to c). It confirms that the value obtained experimentally for the potential step in c) is close to the theoretically expected Nernstian value for a two-orders of magnitude concentration difference.

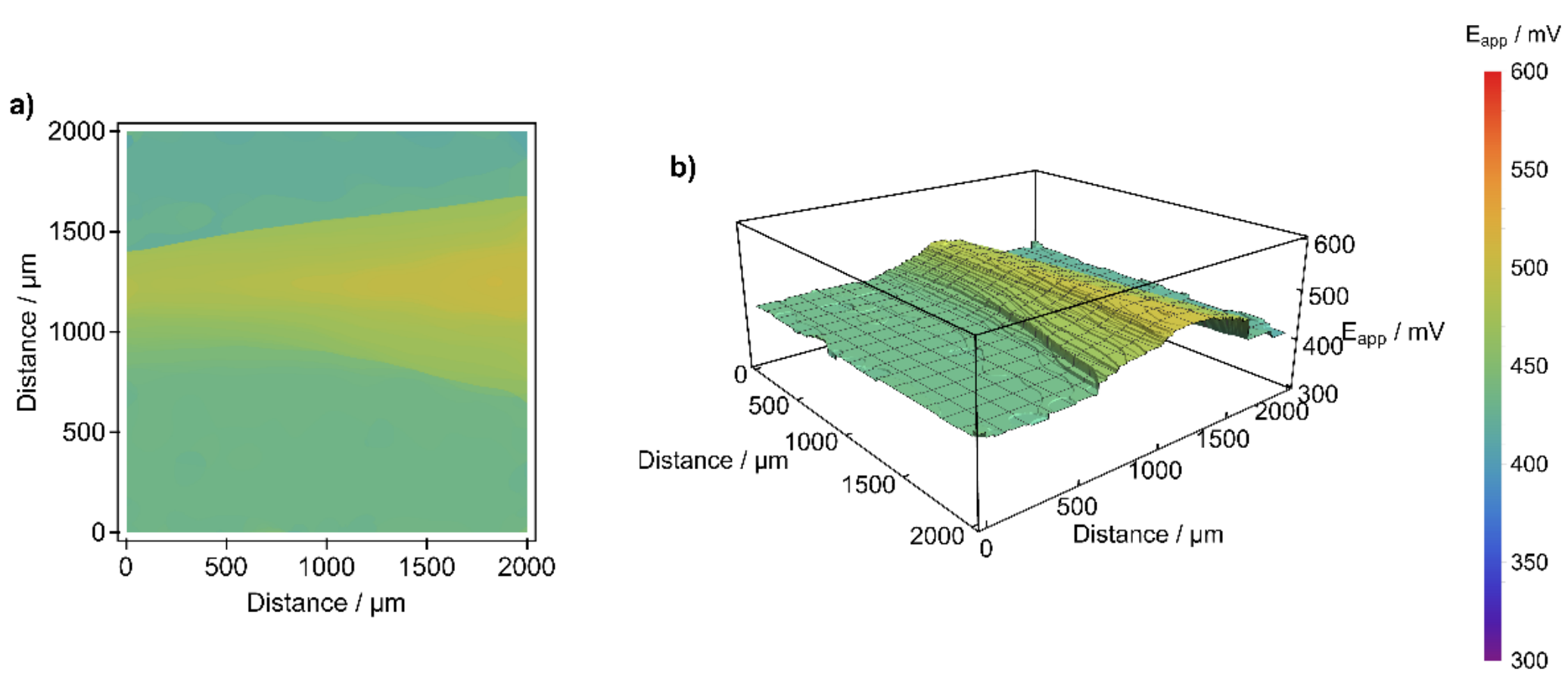

Figure S12. Ammonium production by enzymatic activity of Jack bean urease in the opto-electrochemical flow cell. The upper solution contained the enzyme (100 nM) and the lower solution the substrate (urea, 100 mM). The background electrolyte for both solutions was 25 mM $MgCl_2$. a) Density plot of the peak potential distribution recorded for all pixels in the image. b) Corresponding 3D representation of the potential distibution shown in a).

References

(1) Cesareo, S. D.; Langton, S. R. Kinetic Properties of *Helicobacter Pylori* Urease Compared with Jack Bean Urease. *FEMS Microbiology Letters* **1992**, *99* (1), 15–21. https://doi.org/10.1111/j.1574-6968.1992.tb05535.x.

(2) Krajewska, B.; Ciurli, S. Jack Bean (*Canavalia Ensiformis*) Urease. Probing Acid–Base Groups of the Active Site by pH Variation. *Plant Physiology and Biochemistry* **2005**, *43* (7), 651–658. https://doi.org/10.1016/j.plaphy.2005.05.009.

(3) Dixon, N. E.; Riddles, P. W.; Gazzola, C.; Blakeley, R. L.; Zerner, B. Jack Bean Urease (EC 3.5.1.5). V. On the Mechanism of Action of Urease on Urea, Formamide, Acetamide, *N* -Methylurea, and Related Compounds. *Can. J. Biochem.* **1980**, *58* (12), 1335–1344. https://doi.org/10.1139/o80-181.

(4) Lide, D. R.; Baysinger, G.; Chemistry, S.; Berger, L. I.; Goldberg, R. N.; Kehiaian, H. V. CRC Handbook of Chemistry and Physics.

(5) Gosting, L. J.; Akeley, D. F. A Study of the Diffusion of Urea in Water at 25° with the Gouy Interference Method[1]. *J. Am. Chem. Soc.* **1952**, *74* (8), 2058–2060. https://doi.org/10.1021/ja01128a060.

(6) Zhang, Y.; Hess, H. Enhanced Diffusion of Catalytically Active Enzymes. *ACS Cent Sci* **2019**, *5* (6), 939–948. https://doi.org/10.1021/acscentsci.9b00228.

(7) Mattos, G. J.; Rothen, J. A.; Cherubini, T. J.; Bakker, E. Spatially Resolved Ion Sensing by Voltammetric Ion Transfer Microscopy. *JACS Au* **2025**, *5* (11), 5538–5546. https://doi.org/10.1021/jacsau.5c01034.
